# Dyspnea Measurement in Acute Heart Failure: A Systematic Review and Evidence Map of Randomized Controlled Trials

**DOI:** 10.3389/fmed.2021.728772

**Published:** 2021-10-06

**Authors:** Xiaoyu Zhang, Chen Zhao, Houjun Zhang, Wenjing Liu, Jingjing Zhang, Zhao Chen, Liangzhen You, Yuzhuo Wu, Kehua Zhou, Lijing Zhang, Yan Liu, Jianxin Chen, Hongcai Shang

**Affiliations:** ^1^Key Laboratory of Chinese Internal Medicine of Ministry of Education, Dongzhimen Hospital, Beijing University of Chinese Medicine, Beijing, China; ^2^School of Traditional Chinese Medicine, Beijing University of Chinese Medicine, Beijing, China; ^3^Institute of Basic Research in Clinical Medicine, China Academy of Chinese Medical Sciences, Beijing, China; ^4^Department of Hospital Medicine, ThedaCare Regional Medical Center-Appleton, Appleton, WI, United States; ^5^Department of Cardiology, Dongzhimen Hospital, Beijing University of Chinese Medicine, Beijing, China; ^6^College of Integrated Traditional Chinese and Western Medicine, Hunan University of Chinese Medicine, Changsha, China

**Keywords:** evidence map, systematic review, measurement, acute heart failure, dyspnea

## Abstract

**Background:** Dyspnea is the most common presenting symptom among patients hospitalized for acute heart failure (AHF). Dyspnea relief constitutes a clinically relevant therapeutic target and endpoint for clinical trials and regulatory approval. However, there have been no widely accepted dyspnea measurement standards in AHF. By systematic review and mapping the current evidence of the applied scales, timing, and results of measurement, we hope to provide some new insights and recommendations for dyspnea measurement.

**Methods:** PubMed, Embase, Cochrane Library, and Web of Science were searched from inception until August 27, 2020. Randomized controlled trials (RCTs) with dyspnea severity measured as the endpoint in patients with AHF were included.

**Results:** Out of a total of 63 studies, 28 had dyspnea as the primary endpoint. The Likert scale (34, 54%) and visual analog scale (VAS) (22, 35%) were most widely used for dyspnea assessment. Among the 43 studies with detailed results, dyspnea was assessed most frequently on days 1, 2, 3, and 6 h after randomization or drug administration. Compared with control groups, better dyspnea relief was observed in the experimental groups in 21 studies. Only four studies that assessed tolvaptan compared with control on the proportion of dyspnea improvement met the criteria for meta-analyses, which did not indicate beneficial effect of dyspnea improvement on day 1 (RR: 1.16; 95% CI: 0.99–1.37; *p* = 0.07; *I*^2^ = 61%).

**Conclusion:** The applied scales, analytical approaches, and timing of measurement are in diversity, which has impeded the comprehensive evaluation of clinical efficacy of potential therapies managing dyspnea in patients with AHF. Developing a more general measurement tool established on the unified unidimensional scales, standardized operation protocol to record the continuation, and clinically significant difference of dyspnea variation may be a promising approach. In addition, to evaluate the effect of experimental therapies on dyspnea more precisely, the screening time and blinded assessment are factors that need to be considered.

## Introduction

Dyspnea is the most common presenting symptom among patients hospitalized for acute heart failure (AHF); more specifically, the prevalence of dyspnea at rest was 38.0% in patients in North America and ≥70.1% in patients in the rest of the world (1). There is room for new therapies to improve the symptoms of AHF, given that 36–54.6% of patients do not experience moderate or marked dyspnea relief within 48 h after standard administration (2–5). Moreover, early dyspnea relief is reportedly associated with a better prognosis in patients with AHF (6, 7). Therefore, dyspnea relief constitutes a clinically relevant therapeutic target and endpoint for clinical trials and regulatory approval (8, 9). It is estimated that 46.67% of the clinical trials have used dyspnea as the primary endpoint for the evaluation of treatment efficacy in AHF (10). However, there are still no widely accepted dyspnea measurement standards in AHF.

A narrative review published in 2010 described the strengths and weaknesses of different dyspnea measurement scales in AHF clinical trials, such as the Likert scale, visual analog scale (VAS), Borg scale, and dyspnea severity score (DSS) (8). Likert scales consist of 3-, 5-, or 7-point scales that ask patients to rate their feelings on a categorical spectrum. While the VAS asks patients to report or mark on a 0–100 mm line, and the distance from the 0-level of the scale was measured. The modified Borg scale is a 12-point scale in which words describing increasing degrees are assigned numbers of 0, 0.5, 1, 2, 3, 4, 5, 6, 7, 8, 9, and 10 (11, 12). The DSS was developed specifically to standardize dyspnea measurements in patients with AHF. It consists of asking patients to rate their level of dyspnea on a 5-point Likert scale in each category of provocative movement, which has patients sitting upright with oxygen, sitting upright without oxygen, lying supine without oxygen, walking 50 m as fast as possible, and a post-6-min walk test. The DSS ranges from 1 to 25 and essentially carries out the measurement when patients can no longer progress in performance (13).

Although a decade has passed since then, the best scales of dyspnea measurement in AHF are still not clear, neither are the timing and corresponding effects of measurement. These are of significant importance to the trial design and efficacy evaluation. Therefore, we aim to systematically review and map the current evidence of dyspnea measurement in patients with AHF in randomized controlled trials (RCTs), with the hope to provide some new insights and recommendations for dyspnea measurement.

## Methods

### Information Sources and Search Strategy

This study followed the preferred reporting items for systematic reviews and meta-analyses (PRISMA) reporting guidelines. Guided by the information specialists, two authors conducted a systematic search of the literature in PubMed, Embase, Cochrane Library, and Web of Science databases from inception until August 27, 2020. The search strategy in PubMed is available in eMethods in the Supplementary Material. No language and publication status restrictions were applied. Conference abstract, research protocol, and protocol registration information were screened for further potentially relevant studies. The reference lists of relevant reviews were searched to ensure literature saturation.

### Eligibility Criteria

Two authors independently reviewed the abstracts and retrieved the papers that fulfilled the criteria for closer scrutiny. The inclusion criteria were as follows: (i) The study was an RCT involving human participants with AHF (ii) The dyspnea severity was measured as an endpoint, and (iii) The original research article, conference abstract, research protocol, and registration information were used to identify qualified studies. The exclusion criteria were as follows: (i) repetitive reports of the same study (were included as one study as only) (ii) the measurement of dyspnea was not specified and (iii) the full texts were unavailable. In the event of disagreement, the consensus was achieved through discussion.

### Data Extraction

Data extraction was performed independently by two authors using a designed form which included: first author, year and journal of publication, study design, study sites, trial acronym, intervention, comparison, duration of screening, whether dyspnea was a primary or secondary endpoint, whether dyspnea was a composite endpoint, description of dyspnea measurement, and the timing and results of dyspnea measurement. Any disagreements in data extraction were resolved by discussion.

### Data Analysis and Quality Assessment

For results from more than three RCTs with the same intervention, the dyspnea measurement scale and the timing of measurement were synthesized for meta-analyses using review manager (RevMan5.3, The Cochrane Collaboration, Oxford, UK). For dichotomous outcomes, results were expressed as the risk ratio (RR) with the corresponding 95% confidence interval (CI). For continuous outcomes, results were described with the weighted mean difference (MD) and 95% CI. Heterogeneity was assessed using both the chi-square test (with *P* < 0.10 to indicate significant heterogeneity) and the *I*^2^ value (with *I*^2^ > 50% to indicate significant heterogeneity). Estimates with low heterogeneity (*P* > 0.10 and *I*^2^ < 50%) were pooled using a fixed-effect model. Otherwise, a random effect model was used. All the tests were two-sided, and *P* < 0.05 was considered statistically significant.

The methodological quality for the RCTs was assessed independently by the two authors based on Cochrane risk-of-bias criteria, and each quality item was graded as low, high, or unclear risk. The seven items used to evaluate bias in each trial included the randomization sequence generation, allocation concealment, blinding of participants and personnel, blinding of outcome assessment, incomplete outcome data, selective reporting, and other bias.

## Results

**Figure 1:**
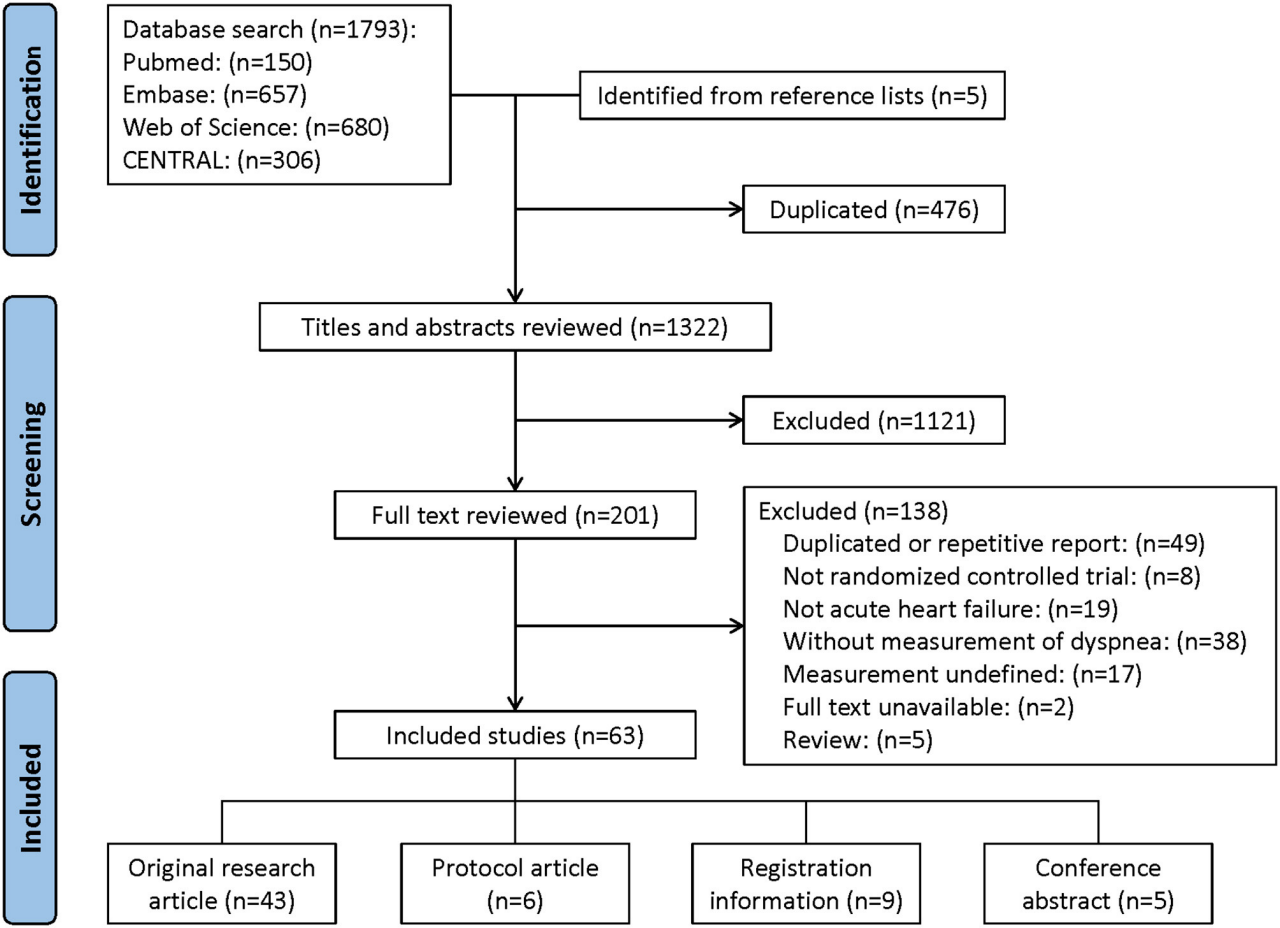
Flowchart for selected studies.

The results of the reference selection and data extraction process are summarized in Figure 1. In all, 1,793 references were identified through database searching and five articles were identified from the reference lists. After a review of titles and abstracts, 201 references were considered potentially eligible and full texts were reviewed. Ultimately, a total of 63 studies were included for data extraction.

### Overview of Dyspnea Measurement

Of the 63 included RCTs, 28 studies used dyspnea as the primary endpoint, and of these, seven studies used dyspnea as the composite endpoint. Of the included studies, 26 studies used dyspnea as the secondary endpoint and nine studies did not specify the primary or secondary endpoint.

The severity of dyspnea was mostly assessed by patients themselves. Physician assessment of orthopnea or dyspnea on exertion was applied in 10 studies, and objective measurements such as pulmonary capillary wedge pressure and peak expiratory flow rate were used in six studies. With respect to the procedure of dyspnea assessment, only five studies described information about supplemental oxygen use, and nine studies described the posture of the patient during dyspnea assessment.

### Dyspnea Measurement Scales

**Figure 2:**
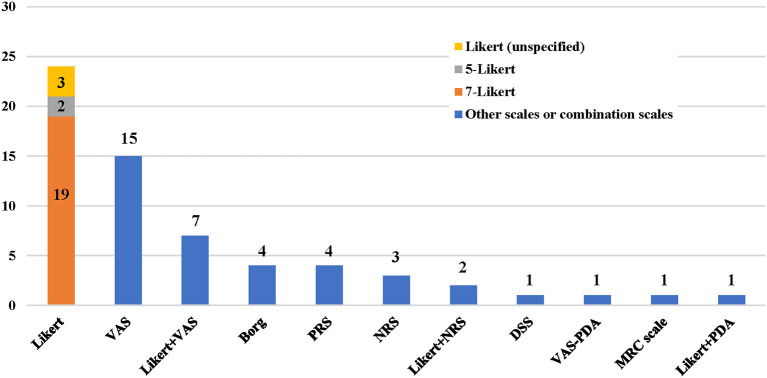
Application frequency of different dyspnea measurement scales. VAS, visual analog scale; PRS, position-based rating scale; NRS, numerical rating scale; DSS, dyspnea severity score; PDA, provocative dyspnea assessment; MRC, medical research council scale.

**Figure 3:**
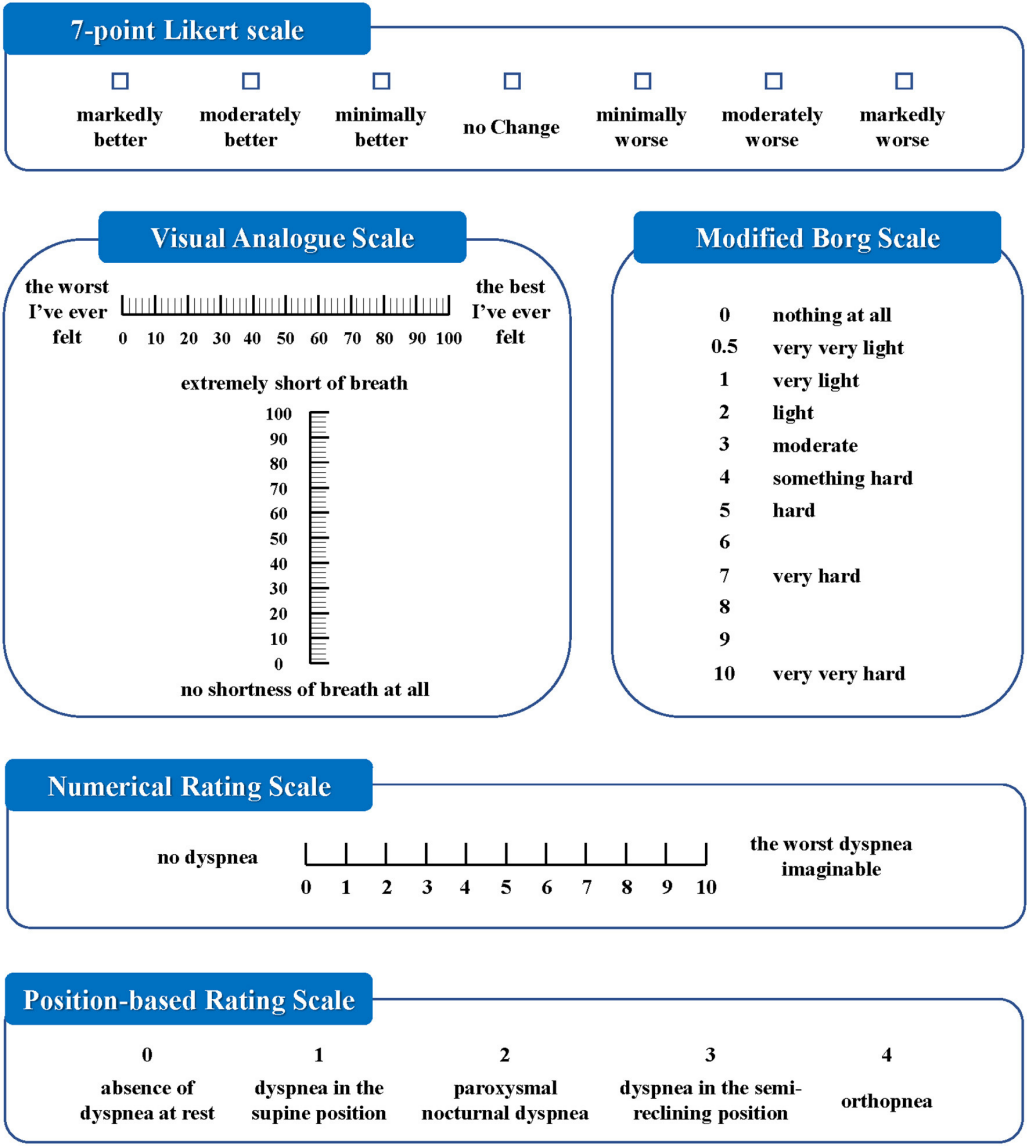
Diagram of dyspnea measurement scales used more than once.

A total of eight dyspnea measurement scales were used in the included studies (Figures 2, 3). The Likert scale was the most widely used measurement scale of dyspnea in patients with AHF, and the 7-point Likert scale accounted for a large proportion. This scale asks patients to rate their level of dyspnea improvement directly on a 7-point categorical spectrum, ranging from “markedly better” to “markedly worse” (14). It can also act as an anchor to identify clinically important differences when used with continuous scales (15).

The VAS, which is understood to sensitively quantify changes in dyspnea severity, is the second-most widely used measurement scale of dyspnea in patients with AHF. The ends of the straight horizontal line are defined as the extreme limits of the parameter to be measured, oriented from the left 0 to the right 100, where 0 was the worst and 100 was the best that the breathing of the patient had ever felt (16). In some studies, the vertical numerical continuum was used, wherein “no shortness of breath at all” was placed at the bottom of the scale and “extremely short of breath” was placed at the top of the scale (17).

The numerical rating scale (NRS) is a segmented numeric version of the VAS in which a respondent selects a whole number from 0 to 10, with 0 being no dyspnea and 10 being the worst dyspnea imaginable (3). A pilot study reported that NRS and VAS showed good agreement when assessing dyspnea severity in the emergency department (18).

Some scales involved statuses such as when a patient experienced dyspnea, respective to the position, provocative movement, and oxygen supply. The position-based rating scale (PRS) assessed dyspnea with the combination of the position and symptom of patients, i.e., absence of dyspnea at rest, dyspnea in the supine position, paroxysmal nocturnal dyspnea, dyspnea in the semireclining position, and orthopnea (19).

The provocative dyspnea assessment (PDA) scale refers to an ordered approach to assess dyspnea across a series of conditions that are increasingly difficult for a patient to tolerate. It may provide a robust profile of dyspnea that is sensitive to change. However, in the RED-ROSE trial, exercise provocation proved to have unacceptable feasibility in the AHF cohort (20). Therefore, some researchers modified it and proposed the VAS-PDA. The subjects assessed their dyspnea severity using VAS in up to three positions as tolerated at each time point, with a score of 0 indicating no dyspnea and a score of 100 indicating very severe dyspnea. Position-1: sitting upright on supplemental oxygen. Position-2: sitting upright off oxygen. Position-3: lying supine off oxygen. Subjects acclimated at each position for 5 min. This created a summed scaled score that ranged from the best dyspnea (0 at all 3 positions = 0) to the worst (100 at all 3 positions = 300) (21).

The medical research council (MRC) scale was developed for grading the effect of dyspnea on daily activities. It comprises five items: 1 (experiencing shortness of breath only during vigorous exercise); 2 (experiencing shortness of breath when walking briskly or ascending a gentle slope); 3 (walking slower than other people their age due to shortness of breath or having to stop to catch their breath even when walking slowly); 4 (stopping to catch their breath after walking <100 m or after a few minutes); and 5 (experiencing so much shortness of breath that they no longer leave the home, or experiencing shortness of breath when getting dressed) (22). In one study, this scale was not sensitive enough for patients with AHF to track responses to therapy during a single hospital stay (8).

### Timing and Results of Dyspnea Measurement

**Figure 4:**
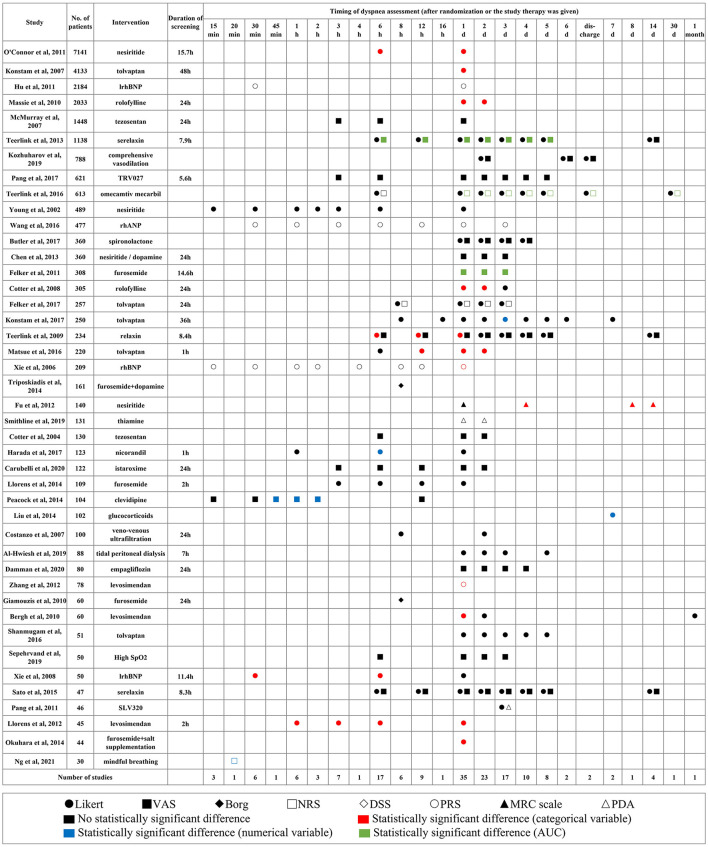
Applied scales, timing, and results of dyspnea measurement in different studies. Shapes represent different scales, colors represent different types of variables, and the statistical significance of results. VAS, visual analog scale; PRS, position-based rating scale; NRS, numerical rating scale; DSS, dyspnea severity score; PDA, provocative dyspnea assessment; MRC, medical research council scale; AUC, area under the curve.

Among the 43 RCTs (3, 4, 14, 16, 17, 19, 21–57) with results reported in original research articles, 23 studies mentioned the duration of screening and in 91.3% of studies, the screening was within 24 h from symptom presentation. The dyspnea was assessed most frequently on days 1, 2, 3, and 6 h after randomization or when the study therapy was given (Figure 4).

Compared with control groups, better dyspnea relief was observed in experimental groups in 21 studies (*p* < 0.05), half of which came from proportions of dyspnea improvement measured by the Likert scale. However, improvements on dyspnea were not consistent when measured by different scales in the same study.

The data from the Likert scale was usually analyzed as a categorical variable considering markedly improved and moderately improved as improvement responders. A few other studies inappropriately analyzed it as a numerical variable and calculated the mean and SD (58). The VAS and NRS were used to quantify persistent relief in dyspnea by the change in area under the curve (AUC) through day 3 or 5.

**Figure 5:**
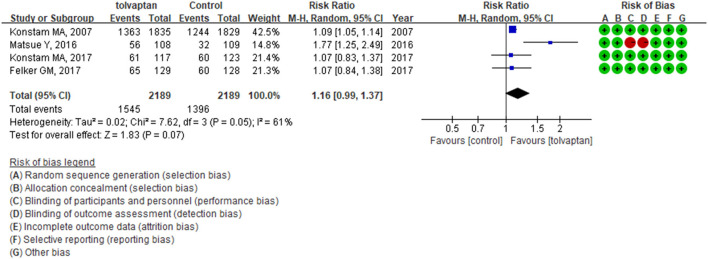
Forest plot comparing tolvaptan vs. control for dyspnea on day 1 and the risk of bias.

Four studies assessed tolvaptan compared with control on the proportion of dyspnea improvement and had divergent results. The synthesized results did not indicate the beneficial effect of tolvaptan on day 1 (RR: 1.16; 95% CI: 0.99–1.37; *p* = 0.07; *I*^2^ = 61%). The EVEREST trial had a much larger sample size, and the AQUAMARINE trial did not apply placebo control and blinding (3, 23). The TACTICS-HF trial and SECRET of CHF trial each produced similar results (Figure 5) (4, 35).

## Discussion

Of the 63 included RCTs, the severity of dyspnea was mostly assessed by patients themselves. Dyspnea is the comprehensive real-world feeling of a patient and deserves full respect. Physician assessment or a single objective measurement is unable to replace the feelings of patients (59, 60); therefore, patient-reported dyspnea measurement scales have been widely used. The Likert scale and VAS were the most accepted tools as demonstrated in this article, which was consistent with the findings of previous reviews (8, 61). Compared with chronic heart failure, the choice of dyspnea measurement scales for patients with AHF emphasizes its usability and sensitivity. The Likert scale is more comprehensible and could directly discriminate the change of dyspnea severity. As a complement, VAS could sensitively quantify the degree of subjective feelings and allow continuous assessment. In the MEASURE-HF trial, the Likert measures of dyspnea initially improved rapidly (day 1, 2) with no significant improvement thereafter (day 7); whereas, the VAS measures of dyspnea improved continually throughout the length of hospital stay (62). Therefore, multiple dyspnea measurement scales should be used simultaneously to capture the entirety of the dyspnea symptom throughout the study.

It is generally believed that a reliable dyspnea measurement with standardized assessment procedures remains a critical unmet need in AHF research (10, 63). Provocative assessment is a reasonable approach, except that exercise provocation has unacceptable feasibility in patients with AHF. However, it is necessary to define the body position and oxygen use of the patient during dyspnea assessment, which was seldom reported in available reports. Furthermore, instead of simply adding up the scores achieved under different conditions, analyzing the scores under separate conditions can make the results more easily understood or interpretable. Regarding the timing of dyspnea measurement, our review showed that it was most frequently on days 1, 2, 3, and 6 h after randomization or when the study therapy was given. It is obvious that the diverse measurement scales, analytical approaches, and the timing of measurements impeded the comprehensive evaluation of the potential therapies. To address this, it is necessary to distinguish the measurement scale and operation procedure for dyspnea assessment. The dyspnea severity and variation could be recorded by unified unidimensional scales, such as the Likert scale and VAS. While the condition and timing of measurement could follow the standardized operation protocol that was established based on the understanding of the disease and experimental therapies (27, 64). With advancements in information technology, we could also record and manage in a timely manner the unstructured data (descriptive text, images, video, and audio material) to understand the provocation condition of dyspnea, its accompanying symptoms, and its impact on the quality of life. This will provide a more general measurement tool to assess patient-reported outcomes like dyspnea.

To more precisely evaluate the effect of experimental therapies on dyspnea in patients with AHF, the screening time from presentation to randomization is one of the factors that should be considered. As is reported in the ASCEND-HF trial, earlier administration of study medication was associated with modestly better dyspnea relief (65). For agents targeting symptom improvements, patients should be enrolled when symptoms are at the peak to minimize concomitant therapy if the effect of the novel agent is to be determined (66). However, the association between earlier administration and better dyspnea relief was not observed on the evidence map and requires further research. In addition, the results of dyspnea assessment can be quite different owing to its subjective nature. In the URGENT study, of the patients with AHF managed conventionally and enrolled within 1 h of first hospital medical evaluation, 58.4% reported moderate or marked dyspnea improvement at 6 h (67). While in the AQUAMARINE study, with a similar screening period, only 13% of the patients with AHF receiving conventional treatment experienced moderate or marked dyspnea improvement at 6 h (4). Therefore, for comparison of treatment effects, it is necessary to conduct a subjective assessment of dyspnea under blind conditions.

### Limitations

This study has some limitations. The recognition of positive results was based on the original reports of the statistically significant difference (*p* < 0.05) which should be interpreted with discretion. Moreover, we only synthesized the results of more than three RCTs with the same intervention, dyspnea measurement scale, and timing of measurement, considering the clinical homogeneity.

## Conclusions

This review and evidence map discusses the current evidence of dyspnea measurement in RCTs with patients with AHF. The applied scales, analytical approaches, and timing of measurement are in diversity, which has impeded the comprehensive evaluation of clinical efficacy of potential therapies managing dyspnea in patients with AHF. A more general measurement tool is warranted, which could be established on the unified unidimensional scales and standardized operation protocol to record the continuation and clinically significant difference of dyspnea variation. With advancements in information technology, we can manage the unstructured data to understand the provocation condition of dyspnea, its accompanying symptoms, and its impact on the quality of life. In addition, to more precisely evaluate the effect of experimental therapies on dyspnea in patients with AHF, the screening time and blinded assessment of dyspnea are factors that should be considered.

## Data Availability Statement

The original contributions presented in the study are included in the article/Supplementary Material, further inquiries can be directed to the corresponding author/s.

## Author Contributions

HS, YL, JC, and XZ: conception and design. HS and JC: administrative support. XZ, CZ, HZ, WL, and JZ: collection and assembly of data. XZ, YL, CZ, KZ, and LZ: data analysis and interpretation. ZC, LY, and YW: provision of study materials or patients. All authors: manuscript writing and final approval of manuscript.

## Funding

This study was supported by the National Key R&D Program of China (2017YFC1700400 to HS) and the National Natural Science Foundation of China (82004219 to XZ and 81803963 to CZ).

## Conflict of Interest

The authors declare that the research was conducted in the absence of any commercial or financial relationships that could be construed as a potential conflict of interest.

## Publisher's Note

All claims expressed in this article are solely those of the authors and do not necessarily represent those of their affiliated organizations, or those of the publisher, the editors and the reviewers. Any product that may be evaluated in this article, or claim that may be made by its manufacturer, is not guaranteed or endorsed by the publisher.
