# Supplementary material for: Dyspnea Measurement in Acute Heart Failure: A Systematic Review and Evidence Map of Randomized Controlled Trials
Source: Front Med (Lausanne). 2021 Oct 6;8:728772. doi: 10.3389/fmed.2021.728772 (PMC8526558; doi:10.3389/fmed.2021.728772)
Supplement: Supplementary file 1 [file Table_1.DOCX]

**eMethods.** Search Strategy in PubMed

#1 ((((((randomized controlled trial[Publication Type]) OR controlled clinical trial[Publication Type]) OR randomized[Title/Abstract]) OR placebo[Title/Abstract]) OR clinical trials[MeSH Major Topic]) OR randomly[Title/Abstract]) OR trial[Title]

#2 humans[MeSH Terms]

#3 #1 AND #2

#4 (((((((((((acute heart failure[Title/Abstract]) OR acute cardiac failure[Title/Abstract]) OR acute coronary failure[Title/Abstract]) OR acute coronary insufficiency[Title/Abstract]) OR acute decompensated heart failure[Title/Abstract]) OR acute destabilised heart failure[Title/Abstract]) OR acute destabilized heart failure[Title/Abstract]) OR acute heart insufficiency[Title/Abstract]) OR acutely decompensated heart failure[Title/Abstract]) OR acutely destabilised heart failure[Title/Abstract]) OR acutely destabilized heart failure[Title/Abstract]) OR ADHF[Title/Abstract]

#5 ((dyspnea[MeSH Terms]) OR dyspn*) OR breathless*

#6 #3 AND #4 AND #5
